# High-grade serous carcinoma with discordant p53 signature: report of a case with new insight regarding high-grade serous carcinogenesis

**DOI:** 10.1186/s13000-018-0702-3

**Published:** 2018-04-27

**Authors:** Yuichiro Hatano, Shinya Fukuda, Hiroshi Makino, Hiroyuki Tomita, Ken-ichirou Morishige, Akira Hara

**Affiliations:** 1 Department of Tumor Pathology, Gifu University Graduate School of Medicine, 1-1 Yanagido, Gifu, 501-1194 Japan; 2 Department of Obstetrics and Gynecology, Gifu University Graduate School of Medicine, Gifu, 501-1194 Japan

**Keywords:** Fallopian tube, Ovary, High-grade serous carcinoma, p53 signature, γ-H2AX

## Abstract

**Background:**

Although p53 signature, benign-appearing epithelial cells with p53 diffuse expression, is frequently found in the fallopian tubes, the clinical and pathological significance of this lesion in the case of high-grade serous carcinoma (HGSC) patients still remains unclear.

**Case presentation:**

A 56-year-old woman was referred to the gynecologist on account of abdominal distention. Since radiological and serological workup suggested that her illness was due to advanced ovarian cancer (FIGO Stage IVB), she received neoadjuvant chemotherapy, and the clinical evaluation of the chemotherapeutic response was a partial response. She underwent total hysterectomy with bilateral salpingo-oophorectomy, omentectomy, and intra-pelvic and para-aortic lymphadenectomy. Histologically, the cancer cells showed high-grade nuclear atypia and spread into the bilateral ovaries, omentum, uterine serosa, and left fallopian tube. The cancer cells showed complete absence of p53 but overexpressed p16, whereas some of benign-appearing tubal epithelial cells overexpressed p53 but lacked p16 expression. The results of direct sequence analysis revealed that the ovarian cancer contains a 1 bp deletion in exon 8 of *TP53*. Finally, the histological diagnosis of HGSC with discordant p53 signature was made. Interestingly, nuclear expression of γ-H2AX, a well-known marker of DNA damage, was not only observed in both p53 aberrantly-expressing lesions but also the benign-appearing tubal epithelium without p53 overexpression. After the histological confirmation, she received adjuvant chemotherapy and has been in disease-free condition without any detectable tumor for 5 months.

**Conclusion:**

Recent evidence suggests that p53 signature is the putative precursor of p53 overexpression-type HGSC. Because the putative precursors of the other p53 immunophenotypical HGSC are not proposed, we presume γ-H2AX-expressing cells without p53 overexpression may be a potent candidate of null-type *TP53*-mutated tubal cells, which are named “γ-H2AX responsive foci.”

## Background

Extra-uterine high-grade serous carcinoma (HGSC) is one of the most lethal malignancies of the female genital tract, and almost always harbors *TP53* mutation [1–4]. Recent evidence indicates that most HGSC cases arise from serous tubal intraepithelial carcinoma (STIC), which is particularly found in the distal fallopian tube [5, 6]. Concurrent STIC and HGSC likely share a common *TP53* mutation and/or p53 expression pattern, indicating that these two serous cancers originate from a single *TP53-*mutated clone [7, 8]. In addition, no intra-tumoral heterogeneity of *TP53* mutation is found in primary untreated HGSC cases [9]. Consistent with this notion, demonstration of identical *TP53* mutation is a useful clue of synchronous lesions [10] and late recurrence [11] of HGSC, and vice versa [12]. Therefore, routine pathological diagnosis of HGSC requires at least p53 immunostaining and extensive investigation of the fallopian tubes, which is based on the SEE-FIM (Sectioning and Extensively Examining of the Fimbriated end) protocol [13].

Such careful pathological examination of the fallopian tubes incidentally detects the p53 signature, which comprises continuous normal-looking tubal epithelium with p53 overexpression. These aberrant p53-expressing cells are occasionally found in asymptomatic healthy women regardless of *BRCA* germline mutation status, and more frequently in women with tubal intraepithelial carcinoma [14]. Interestingly, some of these benign-appearing lesions possess *TP53* mutation identical to the coexisting tubal intraepithelial carcinoma. Considering that p53 dysregulation is believed to be the initiating event for high-grade serous carcinogenesis, the p53 signature is a potent precursor of STIC and/or HGSC.

We herein report the case of a patient with two distinctive p53 aberrantly-expressing lesions that suggest some new insight into the understanding of high-grade serous carcinogenesis.

## Case presentation

### Clinical history

A 56-year-old woman, gravida 2, para 2, was referred to the gynecologist on account of abdominal distention. She had a past medical history of acute pancreatitis, but she had never experienced similar symptoms before. She was on no medications at the time of presentation. She denied familial history of ovarian and/or breast cancer. Blood tests revealed that serum CA125 was high (1520.5 U/mL). Abdominopelvic magnetic resonance imaging showed massive ascites, ovarian masses and numerous nodules in the abdominal and pelvic cavities. In addition, chest computed tomography (CT) scan showed left-supraclavicular lymphadenopathy. Consistent with these radiological findings, positron emission tomography-CT detected fluorodeoxyglucose accumulation within bilateral ovarian masses and left-supraclavicular, peritoneal, para-aortic and intra-pelvic nodules, which were suggestive of distant metastases and peritoneal dissemination from ovarian cancer. Since the clinical diagnosis of advanced ovarian cancer, FIGO Stage IVB (cT3N1M1) was made, three cycles of neoadjuvant chemotherapy (NAC) with paclitaxel/carboplatin were administered. Following NAC, serum CA125 was reduced (97.6 U/mL) on blood tests, all the suspicious tumoral lesions had decreased in size, and the ascites had diminished on radiological re-assessment. The clinical evaluation of chemotherapeutic response yielded a partial response; total hysterectomy with bilateral salpingo-oophorectomy, omentectomy, intra-pelvic and para-aortic lymphadenectomy were performed.

Upon histological assessment of the surgical specimen the ovarian cancer was classified as FIGO Stage IIIB (ypT3bN1MX) [15] and the chemotherapy response score was estimated as 1 (minimal tumor response) [16]. She underwent six cycles of adjuvant chemotherapy with bevacizumab and/or paclitaxel/carboplatin, and has been disease-free without any detectable tumor for 5 months.

### Pathological findings

**Fig. 1:**
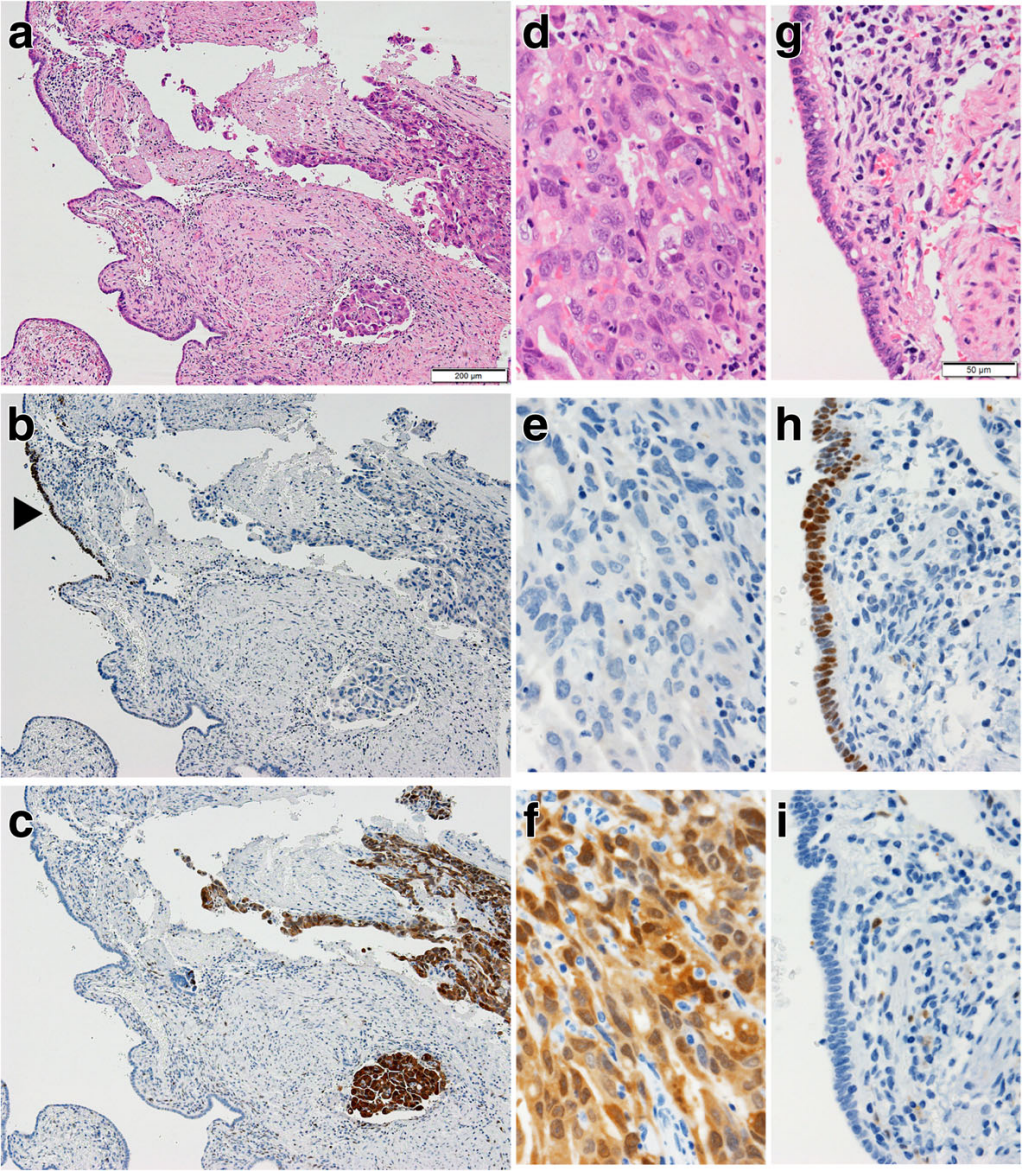
Two distinctive p53 aberrantly-expressing lesions. (**a**)-(**c**) Representative hematoxylin and eosin stained (**a**), p53 (**b**) and p16 (**c**) immunostained images of the left fallopian tube, which contains high-grade serous carcinoma (right) and p53 signature (left, arrowhead). (**d**)-(**f**) Representative hematoxylin and eosin stained (**d**), p53 (**e**) and p16 (**f**) immunostained images of high-grade serous carcinoma. (**g**)-(**i**) Representative hematoxylin and eosin stained (**g**), p53 (**h**) and p16 (**i**) immunostained images of p53 signature

**Fig. 2:**
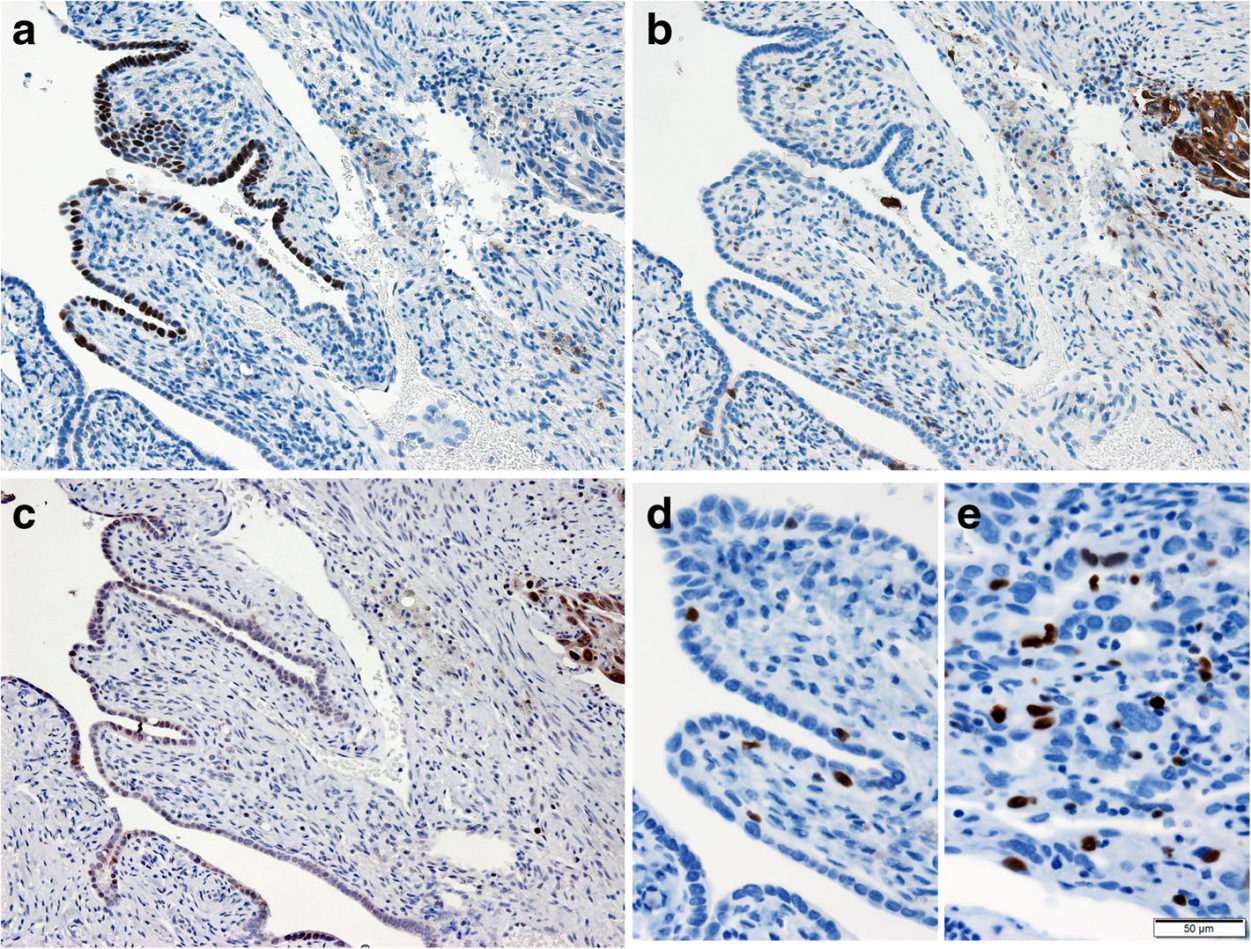
γ-H2AX expression in the distal fallopian tube. (**a**)-(**c**) Representative immunostained images of the p53 (**a**), p16 (**b**) and γ-H2AX (**c**) protein expressions in the high-grade serous carcinoma (right) and p53 signature (left). (**d**)(**e**) Representative Ki-67 immunostained images of p53 signature (**d**) and high-grade serous carcinoma (**e**)

Histologically, the cancer cells showed high-grade nuclear atypia and spread into both ovaries, the omentum, uterine serosa, and left fallopian tube. In the left distal fallopian tube (Fig. 1a-c), these cancer cells (Fig. 1d) showed complete absence of p53 (clone: DO-7; Figs. 1e and 2a), but overexpressed p16 (Figs. 1f and 2b). Interestingly, the benign-appearing tubal epithelium adjacent to the high-grade cancer cells (Fig. 1g) showed an overexpression of p53 (Figs. 1h and 2a), but lacked p16 expression (Figs. 1i and 2b). In addition, nuclear expression of γ-H2AX (clone: ab11174, Abcam, Fig. 2c), was found in both p53 aberrantly-expressing lesions. The Ki-67 labeling index of p53-positive benign-appearing epithelial cells was < 5% (Fig. 2d), whereas that of high grade tumor cells was approximately 15% (Fig. 2e). Finally, we diagnosed these lesions as HGSC with discordant p53 signature.

**Fig. 3:**
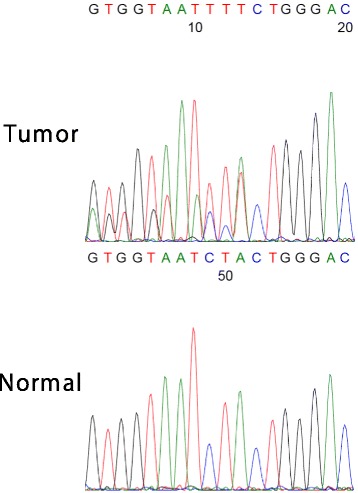
*TP53* mutation analysis of HGSC. A DNA sequence analysis of *TP53* exon 8 from the ovarian and normal tissue with the reverse primer. The sequence of the ovarian tumor shows biallelic pattern from the coding DNA reference number 792, indicating that HGSC harbors a 1 bp deletion (c.792del1) in the *TP53* gene

We also performed *TP53* mutation analysis of HGSC by direct sequencing according to the International Agency for Research on Cancer protocol (http://p53.iarc.fr/Download/TP53_DirectSequencing_IARC.pdf Accessed 22 Dec 2017) with minor modification. Briefly, genomic DNA was extracted from formalin-fixed, paraffin-embedded samples of the ovarian tumor and normal tissue, respectively, using NucleoSpin DNA FFPE XS (Macherey-Nagel, Germany). Exons 4–8 of *TP53* were amplified by polymerase chain reaction and the products were analyzed at the Division of Genomics Research, Life Science Research Center, Gifu University. The results showed that the ovarian cancer contains a 1 bp deletion (c.792del1), which is interpreted as a frameshift mutation, in exon 8 of *TP53*, whereas normal tissue lacked this alteration (Fig. 3).

## Discussion

The patterns of p53 aberrant expression, which is a high-precision predictor of *TP53* mutation, have been traditionally divided into diffuse positive (also known as overexpression), and complete absence (also called as null, diffuse negative) [9]. Intriguingly, the pattern of p53 aberrant expression also predicts the *TP53* mutation status. The overexpression and null patterns are mainly associated with gain-of-function and loss-of-function type *TP53* mutations, respectively. Recently, Kobel et al. reported the cytoplasmic p53 staining, which is the rare third pattern of p53 aberrant expression that arises from genetic mutation in the nuclear localization signaling domain [3]. Collectively, abnormal patterns of p53 expression reflect *TP53* mutation types in HGSC.

In this case, two distinctive p53 aberrantly-expressing lesions were confirmed in the fallopian tube section. Direct sequencing revealed that HGSC harbored a 1 bp deletion in exon 8 of *TP53*. This alteration leads to disruption of the p53 DNA binding domain, which is consistent with the complete absence of p53 expression in HGSC. On the other hand, we failed to investigate *TP53* mutation status of p53 signature because of the insufficient quantity. Given that *TP53* mutation is an initial and central factor in the pathogenesis of HGSC, different *TP53* mutation types likely determine the biological properties of neoplastic cells, including the p53 expression pattern. Thus, discordant p53 aberrantly-expressing patterns are interpreted as the p53 signature being unrelated to the coincident p53 null-type HGSC.

The various p53 immunophenotypes in HGSC implicate the existence of corresponding p53 immunophenotypical precursors. In other words, benign-looking tubal epithelium with abnormal p53 expression includes not only conventional overexpression-type p53 signature but also null- or cytoplasmic staining-type p53 signature (Table 1). Although the conventional p53 signature is detected by routine diagnostic immunohistochemistry, cytoplasmic staining-type p53 signature is probably misdiagnosed as the overexpression-type because of the rare and unknown abnormal pattern. Besides, the other speculative “null-type” p53 signature must be undetectable in the lesion by the same immunohistochemical analysis. Conceptually, these p53 null-type normal-looking tubal epithelial cells should be distinguished from secretory cell outgrowth (SCOUT) [17], which lacks *TP53* mutation.

**Table 1:** Summary of relationship between high-grade serous carcinogenesis and p53 immunophenotype

p53 immunophenotype	Precursor lesion	Malignant form
Diffuse positive (also called overexpression)	p53 signature [14]	HGSC with p53 overexpression, which may reflect GOF p53
Diffuse negative (also called as null, complete absence)	γ-H2AX responsive foci, see Discussion section	HGSC without p53 expression, which may reflect LOF p53
Cytoplasmic staining	Not proposed, but probably misinterpreted as a p53 signature	HGSC with p53 cytoplasmic expression, which is a rare immunophenotype with uncertain clinicopathological significance
Wild-type	SCOUT [17]	HGSC NOS, which shows any p53 immunophenotype

*HGSC* high-grade serous carcinoma, *GOF* gain-of-function, *LOF* loss-of-function, *SCOUT* secretory cell outgrowth, *NOS* not otherwise specified

To demonstrate invisible p53 mutants, we performed additional immunohistochemical analysis of γ-H2AX, a well-known marker of DNA damage [18, 19]. Since the orthodox function of wild-type p53 is activation of the DNA repair system, foci of γ-H2AX-expressing cells may reflect p53 dysfunction and/or *TP53* mutations in the cells. Consistent with a previous report, [14] both the p53 signature and HGSC expressed γ-H2AX. Noteworthy, nuclear expression of γ-H2AX was also detected in the benign-appearing tubal epithelium without p53 overexpression (Fig. 2c). Similarly, Staff et al. reported that γ-H2AX-expressing cells were often found in the fallopian tube but that not all cells overlapped with p53 expression [20]. We speculate that these γ-H2AX-expressing cells without p53 overexpression may be potent candidates for null-type *TP53* mutations, and therefore, named them “γ-H2AX responsive foci.” Interestingly, the Ki-67 labeling index of these cells was almost the same as that of p53 signature. However, it is very difficult to determine whether these candidate cells harbor *TP53* mutation by immunohistochemistry alone, unlike the p53 signature. To identify the hidden precursors of null-type p53 HGSC, other efficient markers of loss-of-function p53 and *TP53* mutation analysis are desired. Taken together, the detection of null-type *TP53* mutation in benign-appearing tubal epithelial cells can help elucidate unknown precursors of STIC and/or HGSC and certainly improve our understanding of microscopic *TP53*-mutated intraepithelial lesions.

Molecular characteristics of HGSC are classified as inevitable *TP53* mutations and diverse gene expression profiles. *TP53-*mutated cells variously evolve by genomic instability, and result in intra- and inter-tumoral heterogeneity in HGSC. These heterogeneities often become a problem in medical practice today [21]. To understand the heterogeneities of prognoses and therapeutic response, molecular classification of HGSC based on gene expression profiles has been attempted [2, 22, 23]. Similarly, p53 expression/*TP53* mutation type in HGSC has shown promise in the classification of the biological characteristics of this lethal high-grade malignancy.

## Conclusions

Recent evidence suggests the precise prediction of *TP53* mutation status solely by p53 immunohistochemistry. This suggestion is also a modest clue to investigate the microscopic lesion, p53 signature. If p53 dysfunction is visualized by other immunohistochemical markers, speculative “null-type” p53 signature could be detected. Therefore, we propose “γ-H2AX responsive foci” as a hidden precursor of null-type p53 HGSC. To progress in the study of the initial genetic event in high-grade serous carcinogenesis, identification of the null- and/or cytoplasmic staining-type *TP53*-mutated normally-appearing cells is required in the future.

## Abbreviations

CT: Computed tomography
FIGO: The International Federation of Gynecology and Obstetrics
HGSC: High-grade serous carcinoma
IARC: International Agency for Research on Cancer
NAC: Neoadjuvant chemotherapy
SCOUT: Secretory cell outgrowth
STIC: Serous tubal intraepithelial carcinoma
